# Plasma lipid abnormalities in Pakistani population: trends, associated factors, and clinical implications

**DOI:** 10.1590/1414-431X20187239

**Published:** 2018-07-23

**Authors:** M. Zaid, S. Hasnain

**Affiliations:** 1 Department of Microbiology and Molecular Genetics, University of the Punjab, Lahore, Pakistan; 2 Department of Life Sciences, University of Management and Technology, Lahore, Pakistan

**Keywords:** Plasma Lipids, Dyslipidemia, Coronary heart disease

## Abstract

Previous studies have reported increased prevalence of coronary heart disease (CHD) in Indians and South Asian settlers in North America. This increased burden of CHD among South Asians is mainly caused by dyslipidemia. To the best of our knowledge, none of the previous works has studied the patterns and prevalence of dyslipidemia in the Pakistani population. The present work aimed to study the plasma lipid trends and abnormalities in a population-based sample of urban and rural Pakistanis. The study included 238 participants (108 males,130 females). Plasma lipid profiles of the participants were determined using standard protocols. We observed that 63% of the study population displayed irregularities in at least one major lipid-fraction including total cholesterol (TC), low-density lipoprotein cholesterol (LDL-C), high-density lipoprotein cholesterol (HDL-C), or triglycerides (TG). The most common form of isolated-dyslipidemia was low HDL-C (17.3%) followed by high TG (11.2%). Several overlaps between high TC, LDL-C, TG and low HDL-C were also noted. Gender, urbanization, and occupational class were all observed to have an impact on lipid profiles. Briefly, male, urban, and blue-collar participants displayed higher prevalence of dyslipidemia compared to female, rural, and white-collar participants, respectively. In comparison to normal subjects, dyslipidemic subjects displayed significantly higher values for different anthropometric variables including body mass index (BMI), body fat percentage, and waist circumference. The present work provides a comprehensive estimation of the prevalence of dyslipidemia and CHD risk in the Pakistani population. This information will be helpful for better healthcare planning and resource allocation in Pakistan.

## Introduction

It has been previously reported that the South Asian population displays a very high prevalence of coronary heart disease (CHD) (1). South Asians tend to develop CHD at younger age in the absence of traditional risk factors (2), according to the "Third Report of the National Cholesterol Education Program (NCEP) Expert Panel on Detection, Evaluation, and Treatment of High Blood Cholesterol in Adults (Adult Treatment Panel III (ATP III)) (3). Moreover, for this population, cardiovascular diseases account for 24% of all deaths among adults (25–69 years) (4). In the last few decades, when the age-adjusted cardiovascular death rates declined in developed countries (5), an alarming increase in the prevalence of cardiovascular diseases has been observed in the Indian population (4,6,7). The growing epidemic of CHD is not specifically observed in South Asia; a rapid rise in CHD burden is also observed in various other developing countries (8–10). This emergence of CHD epidemic in middle- and low-income countries is widely attributed to socio-economic changes, increase in life span and, acquisition of lifestyle-related risk factors. However, it is also noted that CHD prevalence and death rate varies dramatically across the developing world (9) and this variability is attributed to various factors including genetic predisposition, environment, etc. (9).

Dyslipidemia is reported to be closely associated with the pathophysiology of CHD. It is considered as a major, independent, and modifiable risk factor for CHD. Some previous studies suggested that the excess burden of CHD among South Asians is primarily due to dyslipidemia (1). However, very few previous works have studied the prevalence of lipid abnormalities in this population; some focused on the Indian population (7), while others focused on the South Asian immigrants in North America. Nevertheless, to the best of our knowledge, none of the previous studies has performed a detailed assessment of the patterns and prevalence of lipid abnormalities in the Pakistani population.

The present work aimed to estimate the prevalence of plasma lipid abnormalities in the Pakistani population. The prevalence of various types of isolated- and mixed-dyslipidemias were also studied. Moreover, the present work demonstrated the effects of various confounding factors on plasma lipid profile of the study population. CHD-risk according to Framingham risk scoring was also estimated. We also compared the anthropometric characteristics of the dyslipidemic participants with that of the normal participants. To the best of our knowledge, the present study provides the first and most detailed estimation of the prevalence and types of dyslipidemia in the Pakistani population. Our findings could be helpful in proper planning of health care resources for both primary and secondary prevention of CHD in Pakistan.

## Material and Methods

### Participants, study protocols, and ethics

For the present cross-sectional study, we examined 238 healthy volunteers of which 108 were males and 130 females. All the study subjects were above the age of 14 years. Exclusion criteria were self-reported history of bacterial or viral infection, and pregnancy, and patients with the following conditions: on anticoagulation therapy, suffering from bleeding disorder, i.e. hemophilia, low platelets etc., and plastic anemia. Participants with any cancer, and statin users were also not included in the study population because these conditions could interfere with plasma lipid levels. Further exclusion was based on the positive results of diagnostic serological tests of human immunodeficiency virus, hepatitis B virus, and hepatitis C virus. The study protocol was approved by the Ethics Committee of the School of Biological Sciences, University of the Punjab. Informed consent was obtained from each subject before sample collection. In order to obtain basic personal information and medical history, each participant was interviewed and completed a structured questionnaire. Clinical and metabolic characteristics of the study population are reported in Supplementary Tables S1–S3. For age-adjusted analysis, the study population was categorized into the following age groups; G1 (14–22 years), G2 (23–31 years), G3 (32–40 years), G4 (41–49 years), G5 (50–58 years), and G6 (≥59 years). Framingham risk scores (FRS) for CHD over 10 years were calculated, and the study population was categorized into three classes: FRS<10, low ten-year CHD-risk, FRS 10–20, intermediate ten-year CHD-risk, and FRS>20, high ten-year CHD-risk.

The participants of this study were divided into different occupational classes: Blue-collar and white-collar workers, according to Pakistan Standard Classification of Occupations. White-collar workers perform professional, managerial, or administrative work and the activity is performed in an office, cubicle, or other administrative setting. Blue-collar work requires manual labor. Blue-collar workers include those working in skilled trades, craft workers, machine operators, drivers, laborers, agricultural workers, and other manual workers. Students were classified as white-collar participants (Supplementary Table S4). Body weight was measured using a standard analog weighing scale to the nearest kilogram. The measurements for height, waist circumference, hip girth, and wrist circumference were taken to the nearest 0.5 cm using a non-stretchable measuring tape. All of these measurements were taken without shoes, sweater, and jackets. Waist circumference was measured in a section between iliac crests and costal margins at minimal respiration. The hip girth measurements were taken at the level of greatest protrusion of the buttock muscles and the wrist circumference was measured around the widest point. Height, waist circumference, and hip girth measurements were used to calculate waist-to-hip and waist-to-height ratios. Systolic and diastolic blood pressure was measured according to the recommended techniques using a digital sphygmomanometer, and pulse rate was obtained for each participant.

### Sample collection

Intravenous blood was collected from all the subjects after 10±2 h of fasting according to the guidelines of National Committee for Clinical Laboratory Standards (document H18-A4) (11) in vials containing EDTA-anticoagulant agent. Plasma was promptly separated (not more than 4 h after collection of whole blood).

### Determination of plasma lipid levels

Plasma total cholesterol (TC) levels were spectrophotometrically determined using a commercially available kit (Analyticon Biotechnologies AG, 4046, Germany). For the estimation of high-density lipoprotein cholesterol (HDL-C), other lipoprotein fractions were precipitated using HDL-C precipitation reagent (Analyticon Biotechnologies AG, 410, Germany). HDL-C was then estimated using the aforementioned Analyticon kit for the quantitative determination of cholesterol. For estimation of low-density lipoprotein cholesterol (LDL-C) and very low-density lipoprotein cholesterol (VLDL-C), we used an adjustable factor for the triglycerides (TG): VLDL-C ratio as described by Martin et al. (12). HDL-C below 40 mg/dL, LDL-C>129 mg/dL, TG>150 mg/dL, and TC>200 mg/dL were considered abnormal; abnormality status was determined by the criteria given by the expert panel of the NCEP - ATP Final Report (2002) (3).

### Statistical analysis

The results were analyzed by Student's *t*-test and z-test for two population proportions, where applicable. P values <0.05 were considered statistically significant. The data are reported as means±SD unless otherwise indicated.

## Results

### Stratification of the study population into CHD risk-categories

**Figure 1.**
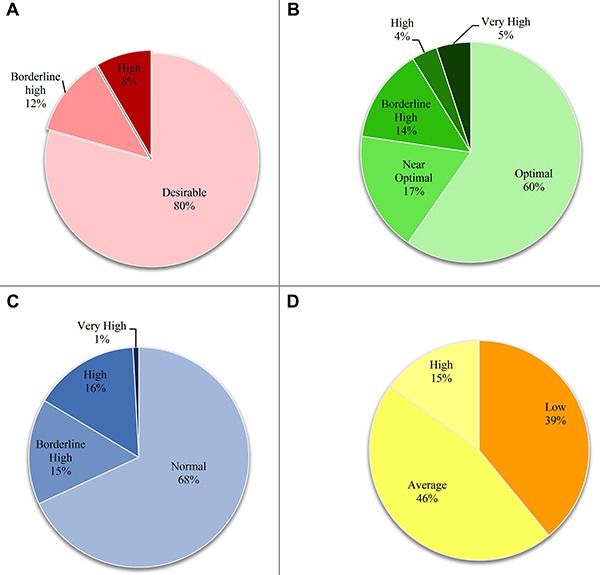
Pie charts displaying the percentage distribution of the study population into various sub-groups according to the plasma levels of *A*: total cholesterol; *B*: low density lipoprotein cholesterol; *C*: triglycerides; *D*: high-density lipoprotein cholesterol. The increasing color intensity within each pie chart signifies increasing coronary heart disease risk.

We first distributed the study population into the various plasma lipoprotein-based CHD risk-categories (Figure 1). It was observed that at least 77% of the population displayed desirable/optimal or near optimal levels of TC and LDL-C (Figure 1A–B), whereas 68% of the population displayed normal levels of TG (Figure 1C). Elevated levels of these plasma lipoprotein subfractions are associated with higher risk of CHD. Less than 10% of the study population was categorized as high- or very high-risk CHD group as depicted by plasma TC or LDL-C levels. Moreover, 17% of the population displayed high or very high levels of TG, hence they were categorized as a high-risk group for CHD.

In contrast, HDL-C levels are inversely correlated with CHD risk. In the present study, we observed that only 15% of the study population displayed high HDL-C levels hence they were categorized as low-risk group for CHD (Figure 1D). On the other hand, 39% of the population was categorized as high-risk CHD group because they displayed low levels of plasma HDL-C. These analyses indicate that the most prevalent type of lipid abnormality in our study population was low HDL-C level.

**Figure 2.**
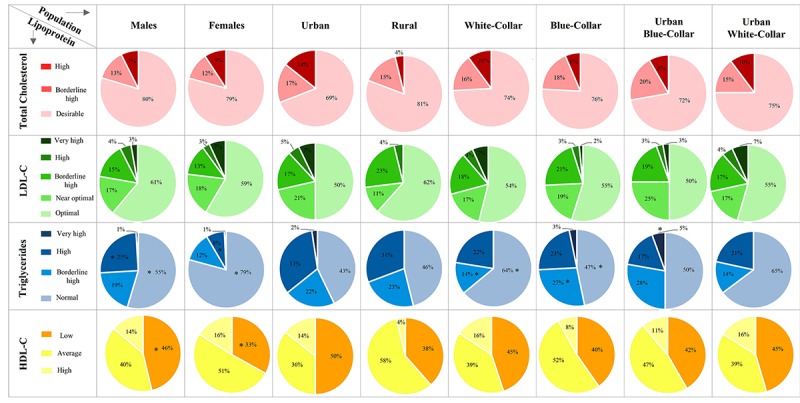
Effect of different confounding factors on stratification of the study population into plasma lipoprotein-based coronary heart disease risk categories according to the ATP-III guidelines. Statistical significance was determined by z-test for two population proportions. *P<0.05. The following comparisons were made: male *vs* female; urban *vs* rural; blue-collar *vs* white-collar; urban blue-collar *vs* urban white-collar. LDL-C: low-density lipoprotein cholesterol; HDL-C: high-density lipoprotein cholesterol.

Concerning the effect of various confounding factors - including gender, age, urbanization, and occupational class - on the stratification of the study population into lipoprotein-based CHD risk-categories, 9% of women had high cholesterol compared to 7% of males, and 7% of women showed high LDL-C compared to 3% of men. An inverse trend was observed for HDL-C, 47% of males had low HDL-C compared to 33% of females with low HDL-C. The percentage of individuals in at least two lipoprotein-based high-risk CHD-categories – i.e. low HDL-C and high TG levels – was significantly higher in the male population (Figure 2) and Supplementary Table S5); 25% of males had high TG and 46% had low HDL-C level. We also compared the absolute values of plasma lipoprotein levels between males and females. These analyses also showed a significant difference in plasma HDL-C and TG levels between males and females (Supplementary Figure S1). Age-dependent analysis revealed that different age groups displayed significant differences in various lipoprotein-based classifications (Supplementary Table S6). High TC was found in 8, 6, 17, 7, 7, 4% in age groups I, II, III, IV, V, and VI, respectively. The absolute values for TC, LDL-C, and TG varied significantly between different age groups (Supplementary Figures S2 and S3), shows age trends for median TC, LDL-C, HDL-C, and TG levels in males and females.

We also compared the effect of urbanization on lipoprotein-based stratification of the study population. As shown in Figure 2, we observed that a high number of rural participants with normal TC and LDL-C levels (81 and 62%, respectively). In addition, low HDL-C levels were more common in the urban subgroup (50%) than in rural population (38%). However, high HDL-C levels were also more common in the urban participants. The major proportion of the rural subgroup displayed average HDL-C levels (58%); hence, they were stratified as average-risk CHD group. The differences between urban and rural sub-groups did not reach statistical significance; nevertheless, the trends indicate that the rural population displayed comparatively fewer irregularities in their plasma lipid profiles. Absolute values of various plasma lipoprotein levels did not display any significant difference between these two groups (Supplementary Figure S4).

In relation to occupational class, the percentage of individuals displaying normal TG levels was significantly lower (47%) in the blue-collar group than in the white-collar group (64%) (Figure 2). Moreover, the percentage of individuals with high HDL-C levels lower in the blue-collar group (8%) than in the white-collar group (16%). However, this difference did not reach statistical significance. Absolute values of various plasma lipoprotein levels did not display significant differences between these two groups (Supplementary Figure S5). It is important to note that the rural group for the present work was composed of only male blue-collar participants. Hence, the observed effects of occupational class in these analyses could have been masked due to the sampling bias. Therefore, we also compared the CHD-risk stratification between blue- and white-collar sub-groups within the urban population (Figure 2). However, the results were the same as for the overall analyses. Absolute values of various plasma lipoprotein levels were not significantly different between these two groups (Supplementary Figure S6).

Previous studies have reported that smoking status may affect the plasma lipid profile (13–15). Here, we did not observe any significant difference between the plasma lipid profiles of smokers and non-smokers (Supplementary Figures S7 and S8).

**Figure 3.**
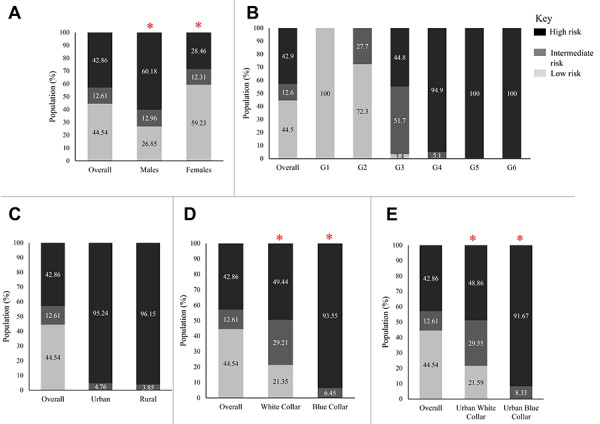
Stratification of the study population into CHD-risk categories according to Framingham Risk Scoring (10-year CHD risk). Comparisons were made between *A*: Males and females; *B*: Different age groups in years (G1: 14–22, G2: 23–31, G3: 32–40, G4:41–49, G5: 50–58, and G5: ≥59); *C*: Urban and rural; *D*: Blue-collar and white-collar; *E*: Urban blue-collar *vs* urban white-collar. Statistical significance was determined by the z-test for two population proportions. *P<0.05, comparison of high-risk groups.

Figure 3A depicts stratification of the study population in different risk categories. We observed that the FRS of 43% of the population was >20%, hence, they were categorized as high-risk group. Significant differences were observed between male and female participants when FRS between these two groups was compared. The percentage of high-risk individuals was significantly higher in the male participants. As expected, FRS tended to increase with age (Figure 3B). Moreover, no significant difference was observed for FRS when urban and rural participants were compared (Figure 3C), whereas, FRS for blue-collar and urban blue-collar groups was significantly higher than white-collar and urban white-collar groups, respectively (Figure 3D,E).

### Prevalence of various forms of isolated- and mixed-dyslipidemias

**Figure 4.**
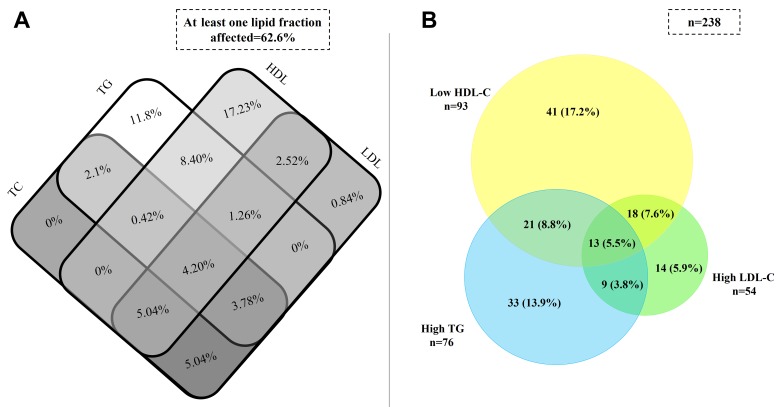
Prevalence of isolated- and mixed-dyslipidemias in the study population. *A*, Venn-Diagram displays overlaps between prevalence of high TC, LDL-C, TG and low HDL-C levels in the study population. *B*, Venn-Diagram displays overlaps between prevalence of high LDL-C, TG and low HDL-C levels in the study population.TC: total cholesterol; TG: triglycerides; LDL-C: low-density lipoprotein cholesterol; HDL-C: high-density lipoprotein cholesterol.

We defined isolated dyslipidemias as irregularities in the plasma levels of only one of the 4 lipoprotein fractions that were examined here – i.e. TC, LDL-C, HDL-C, and TG. On the other hand, mixed-dyslipidemia is the simultaneous occurrence of aberration in two or more of these lipoprotein fractions. Based on the ATPIII criteria high levels of TC, borderline high levels for LDL-C and TG, and low levels of HDL-C were considered anomalous. We observed that 63% of the population displayed an irregularity in at least one major lipid-fraction. Two forms of isolated dyslipidemias were observed, isolated low HDL-C level (17.23%) and isolated high TG level (11.8%) (Figure 4A; Supplementary Table S7). Nevertheless, several overlaps between high TC, LDL-C, TG and low HDL-C were observed.

We sought to determine the prevalence atherogenic lipoprotein phenotype (ALP) or atherogenic lipid triad, which is characterized by increased plasma levels of TG and LDL-C with decreased HDL-C concentrations. Only 5% of the population displayed ALP. In addition to ALP, the incidence of abnormality in only one or two of the lipoprotein fractions of the lipid triad was also demonstrated (Figure 4B). Isolated low HDL-C level (17.2 %) showed the highest prevalence followed by isolated high TG level (13.9 %), whereas 5.9% of the population displayed isolated high LDL-C level. It should be noted that here the definition of isolated dyslipidemia is different from the one used in Figure 4A – that also considers presence or absence of high TC levels.

### Comparison of anthropometric variables and serum lipoprotein levels between dyslipidemic and normal individuals

We compared several anthropometric variables and plasma lipoprotein levels between dyslipidemic and normal individuals. The individuals displaying abnormal levels of at least one lipoprotein fraction were considered dyslipidemic. We observed that female participants with dyslipidemia displayed significantly higher mean values for age, weight, BMI, body fat percentage, total body fat mass, waist circumference, wrist circumference, waist-to-hip ratio, waist-to-height ratio, and diastolic blood pressure (Table 1). Similar trends were observed in male participants except for age, waist-to-hip ratio, and diastolic blood pressure that showed no significant difference between dyslipidemic and normal individuals. Individuals with dyslipidemia displayed significantly higher values for all plasma lipoprotein fractions except HDL-C, which was significantly lower in dyslipidemic individuals (Table 1).

**Table 1.** Anthropometric variables and serum lipid and lipoprotein levels among the healthy and dyslipidemic participants.

Variables	Male participants (n=108)		Female participants (n=130)	
	Healthy (n=30)	Dyslipidemic (n=78)	Healthy (n=59)	Dyslipidemic (n=71)
Age (years)	38.03±17.92	41.82±16.00	28.34±12.1	34.77±15.60*
Weight (kg)	61.69±9.89	71.48±12.9***	56.49±13.30	61.61±11.82*
Height (cm)	169.84±7.55	170.25±6.30	160.58±6.46	158.16±5.55*
BMI (kg/m^2^)	21.36±2.99	24.72±4.60***	21.92±5.09	24.66±4.72**
BMR (Cal/Day)	1476.61±169.39	1551.35±169.32*	1265.82±141.89	1269.68±126.54
Body fat (%)	28.98±6.29	33.88±6.89**	27.42±8.06	32.19±7.92**
Total body fat mass (kg)	18.24±6.05	24.91±8.78***	16.41±9.19	20.62±8.28**
Waist circumference (cm)	83.23±9.19	93.38±15.48**	77.93±13.20	85.02±13.58**
Wrist circumference (cm)	17.06±1.30	17.69±1.29*	15.40±1.82	16.25±1.68**
Waist-to-hip ratio	0.90±0.06	0.93±0.15	0.81±0.081	0.84±0.08*
Waist-to-height ratio	0.49±0.05	0.55±0.09**	0.49±0.08	0.54±0.08***
Systolic BP (mmHg)	122.67±20.86	124.35±16.54	114.17±19.25	121.17±22.74
Diastolic BP (mmHg)	75.03±11.66	80.42±12.87	73.24±11.89	78.34±11.54*
Fasting glucose (mg/dL)	82.19±20.18^#^	92.30±37.22^#^	85.68±34.32^#^	99.26±46.37^#^
TC (mg/dL)	124.83±29.61	173.25±54.23***	142.42±36.03	182.22±55.99***
LDL-C (mg/dL)	51.45±25.96	106.93±49.55***	67.56±34.03	119.31±53.76***
HDL-C (mg/dL)	55.23±13.22	36.93±14.92***	54.47±14.75	37.74±11.72***
VLDL-C (mg/dL)	18.15±3.17	29.39±10.12***	17.39±3.87	25.17±9.79***
TG (mg/dL)	97.16±29.59	191.50±98.33***	87.08±29.66	147.50±93.69***
Non-HDL-C	69.60±26.68	136.32±52.74***	84.95±35.45	144.48±54.10***
TC/HDL-C	2.32±0.55	5.29±2.43***	2.59±0.81	5.34±2.65***
TG/HDL-C	1.86±0.70	5.60±2.86***	1.64±0.74	4.26±2.98***
LDL/HDL-C	0.97±0.50	3.40±2.16***	1.26±0.74	3.59±2.37***
Non-HDL/HDL-C	1.32±0.55	4.29±2.43***	1.59±0.81	4.34±2.65***

Data are reported as means±SD. *P≤0.05; **P≤0.01; and ***P≤0.001, compared to the respective healthy group (Student's *t*-test). # indicates different number of n: 29 and 66, for male healthy and dyslipidemic subjects, and 58 and 63 for female healthy and dyslipidemic subjects, respectively. BMI: body mass index; BMR: basal metabolic rate; BP: blood pressure; TC: total cholesterol; LDL-C: low-density lipoprotein cholesterol; HDL-C: high-density lipoprotein cholesterol; VLDL-C: very low-density lipoprotein cholesterol; TG: triglycerides.

## Discussion

Population surveillance is crucial in monitoring risk factors for CHD; however, in Pakistan there is a paucity of population-level data on lipid levels. Estimates in India indicate that 79% of adults have dyslipidemia (7). No recent comparable Pakistani data have been published. In the present work, we studied the plasma lipid trends and abnormalities in a population-based sample of urban and rural Pakistanis.

First, we studied the stratification of the study population into various CHD-risk categories based on the plasma lipoprotein levels. These analyses revealed that 39% of the study population was high-risk for CHD. According to TC-, LDL-C, and TG-based classification, however, less than 20% were classified as high-risk for CHD.

Gender-based analyses revealed that males displayed significantly higher prevalence of high TG levels and low HDL-C levels than females. Consequently, males were at higher risk of developing CHD compared to females. The FRS also categorized significantly higher percentage of males into high-risk group compared to females. Previous studies also demonstrate that at any given age men are at greater risk for CHD than women (16). Additionally, ATP III guidelines recognize male sex as a non-modifiable risk factor for CHD. Nonetheless, the underlying causes for the gender difference in CHD-risk are not completely deciphered. One possible explanation is the earlier onset of risk factors in men, e.g., elevations of LDL-C and blood pressure, and lower HDL-C. However, the Framingham Heart Study has shown that the differences in absolute risk between the sexes cannot be entirely explained by standard risk factors (16).

ATP III guidelines also include age in the list of non-modifiable risk factors for CHD. CHD-risk increases steeply with advancing age. At any given level of LDL-C, risk for CHD is higher in older than in younger people (16). In the present work, we observed significant differences between different age groups in terms of plasma lipoprotein levels. However, a linear trend between the two factors was not observed. As expected, FRS increased sharply with advancing age.

The increasing burden of CHD in the developing world has also been widely attributed to urbanization. Pakistan is an agricultural country and the life style of the people living in rural areas is vastly different from that in urban areas. There are major differences in the dietary patterns and the level of physical activity between the two populations. In the present study, a higher number of the urban participants displayed abnormal LDL-C and HDL-C levels compared to the rural participants. However, these differences did not reach statistical significance.

Several previous studies have shown a strong relationship between socioeconomic status and CHD (17). It has been observed that CHD morbidity and mortality are higher in lower social classes. Among all the lipid variables, low HDL-C was shown to be most consistently associated with low socioeconomic status (18). In the present study, we observed a significantly higher prevalence of plasma triglyceride abnormalities in the blue-collar group. Occupational-class, income, and education are all considered measures of socio-economic position and have been used interchangeably in previous studies. Recent works suggest that these socio-economic position measures are correlated but they measure different phenomena and with different causal mechanisms. Hence, they should not be used interchangeably as indicators of a hypothetical latent social dimension (19). However, for our study-population, these factors were strongly correlated (data not shown).

Most importantly, we observed that 63% of the population displayed irregularity in at least one major lipid-fraction. The most prevalent form of dyslipidemia was isolated low HDL-C level (17.22%). Previous works have also reported significantly higher prevalence of this novel dyslipidemic profile in Asian population compared to non-Asians (20). However, these studies did not include any Pakistani participants. Studies have demonstrated the significance of isolated low HDL-C level as an independent risk factor for CHD. In this study, after isolated low HDL-C level, the most prevalent form of plasma lipid abnormity was isolated high TG level (11.2%).

We measured lipoprotein ratios as they are indicators of vascular risk and their predictive capability is greater than the isolated parameters. The TC/HDL-C ratio is known as the atherogenic index and the LDL-C/HDL-C ratio is an important predictor of cardiovascular risk. The LDL-C/HDL-C ratio appears to be as useful as the TC/HDL-C ratio, because approximately two-thirds of plasma cholesterol are found in LDL-C, and consequently TC and LDL-C are closely related. The increase in these ratios predicted a greater cardiovascular risk in a wide range of cholesterol or TG concentrations; the risk is significantly higher when triglyceridemia is present (21). In our study, TG level was not higher than 300 mg/dL but LDL-C/HDL-C and TC/HDL-C ratios were significant. The predictive capabilities among isolated parameters and ratios has been compared. TC/HDL-C ratio has high discriminatory power as well as great predictive capacity for coronary heart diseases (22). In this study, the TC/HDL-C ratio was significantly different between healthy and dyslipidemic subjects. We have also calculated the non-HDL-C/HDL-C ratio as non-HDL cholesterol has been recommended as therapeutic target in individuals with high TG concentration and suggested to be a marker of serum apoB concentration (23,24).

To the best of our knowledge, the present study provides the first and most detailed estimation of the prevalence and types of dyslipidemia and the related burden in the Pakistani population. This information will be helpful for better healthcare planning and resource allocation in Pakistan, a country that has a very high burden from CHD and limited health care resources.

## Supplementary Material

Click here to view [pdf].
